# Experiencing malevolent voices is associated with attentional dysfunction in psychotic patients

**DOI:** 10.1111/sjop.12024

**Published:** 2013-04

**Authors:** BODIL KRÅKVIK, TORE STILES, KENNETH HUGDAHL

**Affiliations:** 1 St. Olavs Hospital; Nidaros DPS, Research and DevelopmentTrondheim, Norway; 2 Norwegian University of Science and Technology, Department of PsychologyTrondheim, Norway; 3 Department of Biological and Medical Psychology, University of BergenBergen, Norway; 4 Division of Psychiatry, Haukeland University HospitalBergen, Norway; 5 Department of Radiology, Haukeland University HospitalBergen, Norway

**Keywords:** Attentional impairment, auditory hallucination, depression, psychosis, schizophrenia

## Abstract

Inattention in people with schizophrenia is common. However, there has been little research on the association between inattention and auditory hallucinations. The aim of the study was to investigate how inattention is affected by beliefs about voices as benevolent and malevolent and perceived control of voices. A total of 31 patients who experienced auditory hallucinations and who met the criteria for schizophrenia or other psychosis completed the attention subscale of the Scale for the Assessment of Negative Symptoms (SANS) and the Connors’ Continuous Performance Test II (CCPT-II). The revised Beliefs About Voices Questionnaire (BAVQ-R) was used to assess malevolent and benevolent beliefs about voices, and severity of auditory hallucinations (the Psychotic Symptom Rating Scales; PSYRATS) was used to assess perceived control of voices and frequency of voices. Levels of depression (the Beck Depression Inventory; BDI), anxiety (the Beck Anxiety Inventory; BAI), severity of overall psychiatric symptoms (the Brief Psychiatric Rating Scale; BPRS), and severity of negative symptoms (SANS) were assessed to control for their potential confounding effects. The relations between the variables were explored with correlations and multiple hierarchical regression analyses. The results indicated that more malevolent, but not more benevolent, beliefs about voices predicted lower levels of attention, independently of general psychiatric symptoms and various other psychotic symptoms such as frequency of and perceived control of voices. These findings suggest an important relationship between malevolent beliefs about voices and levels of inattention. The possible impact of changing beliefs about voices to improve attentional functioning is discussed.

## INTRODUCTION

Schizophrenia is associated with positive symptoms such as auditory hallucinations and delusions (Frith, 1993), as well as a wide variety of cognitive impairments (Bozikas, Andreou, Giannakou *et al.*, 2005). Cognitive impairment, especially attentional dysfunction, is often so severe that it interferes with activities such as personal care skills, work and interacting with others (Kern, Green & Satz, 1992). Moreover, it may sometimes even preclude treatment benefits (Green, Kern, Braff & Mintz, 2000). It has been demonstrated that patients diagnosed with schizophrenia have difficulties in allocating attentional resources to the appropriate stimulus sources (Gourovitch & Goldberg, 1996; Hugdahl, 2009). Compared with healthy controls, patients diagnosed with schizophrenia show weak performance on cognitive tests that measure vigilance, which refers to distraction and reduced ability to maintain attention over time. Further, patients diagnosed with schizophrenia have lower scores than controls on tests based on selective attention (Egeland, Rund, Sundet *et al.*, 2003; Oltmanns & Neale, 1975) as well as other cognitive functions (Egeland, Sundet, Rund *et al.*, 20032003b; Løberg, Hugdahl & Green, 1999). In addition, it has been demonstrated that positive symptoms may be associated with poor performance on tests of auditory attention, which may suggest a dysfunction within neural networks underlying attentional processes (Berman, Viegner, Merson, Allan, Pappas & Green, 1997; Hugdahl, Løberg & Nygård, 2009). Recently, it has been shown that attentional dysfunction in schizophrenia can be meaningfully rated and interpreted with clinical ratings from the attentional subscale of the Scale for the Assessment of Negative Symptoms (SANS; Vadhan, Serper, Harvey, Chou & Cancro, 2001) tapping a global rating of inattention.

Approximately 70% of individuals diagnosed with schizophrenia experience auditory hallucinations (Nayani & David, 1996). Most of them find the experience of auditory hallucinations distressing, annoying, disabling, and incriminating (Chadwick & Birchwood, 1994; Leudar, Thomas, McNally & Glinski, 1997). For some patients auditory hallucinations may be experienced as positive, giving them strength and enhancing their self-esteem (Romme & Escher, 1989).

According to Beck and Emery’s (2005) cognitive theory, psychiatric disorders and symptoms are related to cognitive processes, where negative thoughts are believed to result from the activation of underlying negative beliefs that are directed towards stimuli that are perceived as threatening.

A translation of the Beck and Emery’s (2005) theory to the experience of voice hearing suggests that the voice hearer’s cognitive appraisal system might be activated by the stimulus of the voice, which in turn might influence the level of attention to other more relevant external stimuli.

A possible way to interpret the relevance of Beck and Emery’s theory for auditory hallucinations is to consider the identification of the voice as either malevolent or benevolent as primary appraisal concerns, while whether or not the hearer believes he or she can cope with the voice can be considered secondary appraisal (Lazarus & Folkman, 1984). It should be pointed out, however, that these are theoretical assumptions that need empirical support since it is also plausible that perceptions of degree of perceived control of the voice influence the perception of the content of the voice, that is, that primary and secondary appraisals are reversed. For example, if the voice is considered malevolent and the patient underestimates his or her ability to cope with the voice, the patient might feel threatened, which will result in an increased attentional shift towards the threat. An attentional shift towards monitoring the possibility of hearing a voice may result in impairment in paying attention to other environmental stimuli and for the execution of parallel tasks. It is reasonable to assume that the result of these two cognitive appraisals may affect a patient’s attentional system.

Moreover, for those who experience malevolent voices, lack of perceived control has been reported (Honig, Romme, Ensink, Escher, Pennings & deVries, 1998; Hugdahl *et al*., 2009), and also that perceived control over a voice is a characteristic that distinguishes individuals who experience less frequent auditory hallucinations from those who experience more frequent and distressing auditory hallucinations (Leudar *et al.*, 1997). Hence, it is not unreasonable to expect that perceiving a voice as malevolent, combined with low ability to control the voice, will affect the hearer’s level of attention.

To our knowledge no studies have examined to what extent the beliefs about voices affect attentional functioning in patients with auditory hallucinations. Accordingly, the aim of the study was to examine the extent to which the value attributed to voices as malevolent and benevolent and also the control of the voice respectively predict levels of attention to environmental stimuli as measured by the SANS attentional subscale when controlling for other potential psychotic and non-psychotic psychiatric symptoms. More specifically, it was predicted that malevolent beliefs about voices would be significantly related to level of attentional function, while benevolent beliefs about voices would not be related to level of attentional function since benevolent voices do not elicit a need for attentional control to the same degree as malevolent voices. It was also predicted that level of perceived control of voices would be significantly related to level of attentional function.

Research aiming to examine the predictive role of cognitive processes related to level of attentional function should ideally control for the potential effects of negative symptoms (cf. Ventura, Hellemann, Thames, Koellner & Neuchterlein, 2009), other psychotic symptoms and the overall severity of psychiatric symptoms (cf. Birchwood & Chadwick, 1997; Pallanti, Quercioli & Hollander, 2004). Thus, measures of these factors were included as control procedures. We predicted that negative symptoms would not be related to level of attentional function, since this should be specifically related to positive symptoms and auditory hallucinations.

## METHOD

The study was part of a larger randomized control trial (RCT) in mid-Norway, comparing cognitive therapy in addition to treatment as usual (TAU) with TAU for patients with drug-resistant auditory hallucinations and delusions. Data for the current study were collected during the period 2002–2005. The study received approval from the Regional Committees of Medical and Health Research Ethics in the Middle of Norway and the Norwegian Social Science Data Services.

### Procedure

Participants were recruited through consultant psychiatrists, clinical psychologists and psychiatric nurses from outpatient and inpatient mental health clinics. The assessments were administered and scored by independent interviewers, who had been trained in interview skills and scoring procedures by senior researcher Rolf Wilhelm Gråwe. The interviewers met regularly in order to prevent deviances in accuracy of ratings during the course of the study. All of the interviewers had clinical experience in treating people with psychosis. The interviewers were blind to the hypotheses tested in this study. The Connors’ Continuous Performance II (CCPT-II) was administered by a licensed psychologist. The participants completed the various tests before randomization to the trial conditions.

## SUBJECTS

Participants were entered into the trial if they had experienced auditory hallucinations within the last six months and if they had caused distress despite the use of antipsychotic medication. In addition, they had to meet the following criteria: (1) diagnosed in accordance with the International Classification of Diseases, 10th Revision (WHO, 1992) (ICD-10) as having either schizophrenia, schizoaffective disorder, or persistent delusional disorder; (2) were in the age group 18–60 years; and (3) were able to give written consent to participate in the study. A diagnosis of substance abuse was an exclusion criterion.

Out of 68 patients referred to the RCT study, 9 patients were excluded because they were not in the target group, 9 withdrew before the baseline measurement, 2 did not meet for baseline measurement, 2 did not complete the baseline measurement, and 1 was unable to speak Norwegian sufficiently well. A total of 45 patients were thus included in the RCT study. Of these, 31 experienced auditory hallucinations, and were consequently included in the present study.

The mean age of the participants was 36.6 years (*SD* = 10.7; range: 19–59 years). In total, 17 (54.8%) were male and 14 (45.2%) were female. At their first lifetime contact with the mental health care system they were 21.3 years of age (*SD* = 7.4; range: 4–36). Their average age at the first hospitalization was 25.6 years of age (*SD* = 6.5; range: 13–39). Two of the subjects had never been admitted to a psychiatric hospital. In total, 25 (78.1%) of the participants were single, 22 (70.7%) were outpatients, and all were receiving antipsychotic medication. In addition, 27 (87.1%) of the participants met the criteria for schizophrenia, 3 (9.7%) met the criteria for persistent delusional disorder, and 1 (3.2%) met the criteria for schizoaffective disorder. The sample had been hearing voices for an average of 12.4 years (*SD* = 9.9; range: 1–40). The schizoaffective and delusional disorder patients were included because of persistent auditory hallucinations and the overlap in general symptomatology between these diagnostic disorders that are all psychotic disorders.

### Instruments

The revised Beliefs About Voices Questionnaire (BAVQ-R; Chadwick, Lees & Birchwood, 2000).  The BAVQ-R comprise 35 items that generate five scales. Three subscales measure beliefs about the voice’s malevolence, benevolence and omnipotence, and two scales are concerned with the participant’s behavioral and affective responses to the voice. Each item was rated on a 0 to 4-point Likert scale (disagree (0), unsure (1), agree slightly (2), agree strongly (3)). For the purpose of the study, only data from the malevolent (BAVQ-R-MAL) and benevolent (BAVQ-R-BEN) subscales were used. Internal reliability for the translated version (into Norwegian) was 0.67 for the BAVQ-R-MAL and 0.85 for the BAVQ-R-BEN.

*The Psychotic Symptom Rating Scales (PSYRATS;* Haddock, McCarron, Tarrier & Faragher, 1999). PSYRATS measure the severity of a number of dimensions of auditory hallucinations and delusions (two separate scales), including the amount and intensity of distress associated with these symptoms. A five-point ordinal scale (0–4) is used to rate symptom scores. The auditory hallucinations subscale (PSYRATS-AHS), an 11-item scale, was used in the present study. It has been shown to possess acceptable inter-rater reliability and validity (Haddock *et al.*, 1999).

*The attention subscale of the Scale for the Assessment of Negative Symptoms (SANS;* Andreasen, 1989). SANS was used to rate attentional dysfunction. The SANS is an interview scale to assesses symptom complexes in obtain clinical ratings of negative symptoms in patients with schizophrenia. The attention subscale consists of social inattentiveness, inattentiveness during mental status testing and global rating of inattentiveness. A global rating of inattentiveness was used in the present study. Higher scores indicate higher levels of inattention. The subscale has been shown to possess excellent inter-rater reliability (Andreasen, 1982) and acceptable concurrent and discriminant validity (Vadhan *et al.*, 2001). Moreover, it has been demonstrated to be significantly associated with specific neuropsychological tests of attention, but not working memory or executive functioning (Basso, Nasrallah, Olson & Bornstein, 1998; Vadhan *et al.*, 2001).

*The Connors’ Continuous Performance Test – Second Edition (CCPT II;* Connors & Staff, 2000). CCPT II is a computerized test designed to measure sustained attention and vigilance. Data from the CCPT II test were analysed with the Detectability (d’) measure included in the test, since it allows for assessment of both commission and omission hits and errors. Higher scores indicate higher levels of inattention. The test was included to examine to what extent it correlates with inattention measured by the attention subscale of SANS. The participants were given instructions to press a key immediately after each letter presented on the screen except “X.” The test consists of six blocks and each block is made up of three sub-blocks. The letters are presented at varying speeds (1, 2 or 4 seconds intervals) – the order of the sub-blocks vary from block to block. The test takes approximately 14 minutes to complete.

*The Assessment of Negative Symptoms (SANS;* Andreasen, 1989). Four subscales of the Scale were used to assess negative symptoms. The four subscales are: affective flattening or blunting; alogia; avolition-apathy; and anhedonia-asociality. The SANS is a six-point (0–5) rating instrument. The global score of the four subscales was used. Higher scores indicate more negative symptoms. The four subscales have been demonstrated to have excellent inter-rater reliability (Andreasen, 1982) and satisfactory concurrent and discriminant validity (Vadhan *et al.*, 2001).

*The Brief Psychiatric Rating Scale (BPRS;* Ventura, Green, Shaner & Liberman, 1993). BPRS was used to measure the severity of general psychiatric symptoms. The BPRS measures severity of 24 different psychiatric symptoms on one of seven ordinal intensity descriptors ranging from low (not present or not observed) to extremely severe. Item 9 (hallucinations) was excluded to prevent overlap with the other measures tapping auditory hallucinations. The BPRS has been shown to be a sensitive measure of psychiatric symptoms with good inter-rater reliability (Ventura *et al.*, 1993).

*The Beck Depression Inventory (BDI;* Beck, Rush, Shaw & Emery, 1979). The BDI is a 21-item, 4-point self-rating scale to assess the severity of depression. It has extensively been shown to be a reliable and valid measure of syndrome depression severity in both clinical and nonclinical populations (Beck, Steer & Garbin, 1988).

*The Beck Anxiety Inventory (BAI;* Beck & Steer, 1990). The BAI is a 21-item self-report instrument that measures anxiety severity for the past week, including the day of completion. The BAI is established as a reliable and valid measure, and is recommended as a companion instrument to the BDI, particularly for individuals with comorbid depression and anxiety (Beck & Steer, 1993).

### Data analysis

The statistical analyses were carried out using SPSS-15. To investigate concurrent validity, Pearsons’s correlation analysis was used to examine correlations between the measures. Separate hierarchical multiple regression analyses were performed to examine to what extent beliefs about voices and perceived control of voices could predict level of inattention as measured by the SANS attention subscale. Since this was an exploratory study with a low sample size, an alpha level of 0.05 was considered statistically significant.

## RESULTS

The correlational analyses (Table 1) revealed that the SANS attention subscale was significantly correlated with the Detectability (d’) measure of the Connors’ Continuous Performance Test (CCPT-II). Neither age nor sex was related to the two measures of inattention. However, level of inattention measured with the SANS attention subscale was both positively and significantly associated with the severity of general psychiatric symptoms (BPRS), the auditory hallucinations subscale (PSYRATS-AHS), frequencies of the voices, perceived control of the voices, and depression (BDI), but not with negative symptoms measured with the SANS and anxiety (BAI). As predicted, the SANS attention subscale was positively and significantly correlated with the beliefs about voices as malevolent (BAVQ-R-MAL), but not with the beliefs about voices as benevolent (BAVQ-R-BEN). Further, the results revealed that Detectability (d’) was significantly correlated with BAVQ-R-MAL and frequencies of voices.

**Table 1:** Means (M) and standard deviations (SD) and Pearson Correlations for the various measures at baseline (N = 31)

Measure	M (*SD*)	SANS Attention Subscale	CPT II Detectablity (d’)
SANS Attention subscale	1.39 (1.20)		
CCPT-II Detectability (d’)	51.96 (9.18)	0.550**	-
SANS	2.29 (2.31)	−0.030	0.221
BPRS	45.13 (10.21)	0.365*	0.210
PSYRATS - AHS	27.93 (6.79)	0.505**	0.406
Frequencies of voices	2.71 (1.10)	0.566**	0.469*
Control of voices	2.84 (1.18)	0.443*	0.161
BAI	21.96 (12.41)	0.125	0.090
BDI	20.46 (10.26)	0.381*	−0.107
BAVQ-R-BEN	4.28 (4.49)	−0.177	−0.250
BAVQ-R-MAL	9.90 (4.72)	0.641**	0.414*

* *p* < 0.05.

** *p* < 0.01.

Abbreviation: The attention subscale from SANS: Assessment of Negative Symptoms; Detectability (d’) from CPT II: Conners’ Continuous Performance Test – Second Edition; SANS global score without the attention subscale. BPRS: Brief Psychiatric Rating Scale without item 9 (hallucinations); PSYRATS-AHS: Psychotic Symptom Rating Scales for Auditory Hallucination; BAI: Beck’s anxiety scale; BDI: Beck’s depression scale; BAVQ-R-BEN (benevolent) and BAVQ-R-MAL (malevolent) from BAVQ-r: Beliefs About Voices Questionnaire – revised.

### Predictive validity of the voice’s malevolence

Since the BPRS, the PSYRATS-AHS and the BDI were significantly associated with the SANS attention subscale, the effects of these variables were statistically controlled for by entering them in the first three steps of a hierarchical regression analysis. In the fourth step the BAVQ-R-MAL was entered. Scores on the SANS attention subscale was the dependent variable. The results of the regression analysis are summarized in Table 2.

**Table 2:** *Hierarchical regression analysis predicting levels of inattention measured by the SANS attention subscale controlling for BPRS, PSYRATS, BDI and BAVQ-R-MAL* (N = 31)

Variable	Beta	t	p
Step 1			
BPRS	0.365	2.112	0.043
Step 2			
BPRS	0.200	1.156	0.257
PSYRATS-AHS	0.428	2.478	0.019
Step 3			
BPRS	0.054	0.274	0.786
PSYRATS-AHS	0.434	2.565	0.016
BDI	0.272	1.480	0.151
Step 4			
BPRS	0.157	0.927	0.363
PSYRATS-AHS	0.257	1.680	0.105
BDI	0.083	0.500	0.621
BAVQ-R-MAL	0.506	3.378	0.002

*Notes*: BPRS: Brief Psychiatric Rating Scale without item 9 (hallucinations); PSYRATS-AHS: Psychotic Symptom Rating Scales for Auditory Hallucination; BDI: Beck’s depression scale and BAVQ-R-MAL (malevolent) from BAVQ-R: Beliefs About Voices Questionnaire – revised.

The results from step 3 of the regression analysis indicated that neither level of depression nor level of overall psychiatric symptoms predicted level of inattention when scores from the PSYRATS-AHS were in the equation, yet the latter did. More interestingly, when scores from the BAVQ-R-MAL were entered into the equation in step four, only the BAVQ-R-MAL predicted level of inattention. Then the scores of PSYRATS-AHS no longer predicted level of inattention.

Since both the subscale frequency of voices and perceived control of voices from the PSYRATS-AHS were significantly correlated with level of inattention measured with the SANS attention subscale, a new hierarchical regression analysis was conducted substituting the PSYRATS-AHS total score with the scores from the subscale frequency of voices and the scores from the subscale perceived control over the voices. In this regression analysis, BPRS was entered in the first step, frequency of voices in the second step, control of voices in the third step, and finally the BAVQ-R-MAL in the fourth step. The results are summarized in Table 3.

**Table 3:** *Hierarchical regression analysis predicting levels of inattention measured by the SANS attention subscale controlling for two subscales of the PSYRATS-AHS and BAVQ-R-MAL (N* = *31)*

Variable	Beta	t	p
Step 1			
BPRS	0.365	2.112	0.043
Step 2			
BPRS	0.309	2.130	0.042
Frequencies of voices	0.534	3.674	0.001
Step 3			
BPRS	0.297	2.048	0.050
Frequencies of voices	0.442	2.657	0.013
Control of voices	0.188	1.127	0.270
Step 4			
BPRS	0.265	2.109	0.045
Frequencies of voices	0.318	2.141	0.042
Control of voices	0.078	0.529	0.601
BAVQ-R-MAL	0.488	3.190	0.004

*Notes*: BPRS: Brief Psychiatric Rating Scale without item 9 (hallucinations); Frequencies and control of voices from PSYRATS-AHS: Psychotic Symptom Rating Scales for Auditory Hallucination and BAVQ-R-MAL (malevolent) from BAVQ-R: Beliefs About Voices Questionnaire – revised.

The results from step four of the regression analysis indicated that the BPRS, the frequency of voices and the BAVQ-R-MAL significantly predicted level of inattention independently of each other, while levels of perceived control over the voices was not associated with level of inattention.

## DISCUSSION

As predicted, beliefs about voices as malevolent, but not benevolent, were significantly associated with level of inattention measured with the SANS attention subscale, independently of both overall psychiatric symptoms (BPRS) and the severity of auditory hallucinations, such as frequency and perceived control of voices (PSYRATS-AHS). Moreover, neither overall psychiatric symptoms measured by BPRS nor the severity of auditory hallucinations measured by PSYRATS-AHS affected attentional functioning when the effects of malevolent voices were statistically controlled for. However, when the frequencies and perceived control of voices measured by the PSYRATS-AHS were entered in a new regression analysis, then the overall psychiatric symptoms measured by BPRS, the frequencies of voices and levels of malevolent voices significantly and independently predicted level of inattention. As predicted, negative symptoms, as measured by SANS, were not significantly associated with levels of attentional function. Although levels of perceived control of voices was significantly correlated with levels of attentional function, it was not when levels of overall psychiatric symptoms and frequency of voices was statistically controlled for. The latter finding is consistent with earlier research indicating a relationship between frequency of auditory hallucination and levels of perceived control over voices (Honig *et al.*, 1998; Hugdahl *et al*., 2009; Leudar *et al.*, 1997). Thus, the results suggest that cognitive beliefs about voices as malevolent might play a key role in maintaining inattention in schizophrenia and psychosis. This finding has some parallels with a case study reported by Hatashita-Wong and Silverstein (2003), where patients’ inattention was found to be correlated with disabling voices. However, in contrast to the current study, the study conducted by Hatashita-Wong and Silverstein (2003) did not define the voice as either malevolent or benevolent.

A cornerstone in several cognitive models of anxiety (Beck & Clark, 1997; Wells, 1997) is the shift in the direction of attention towards threatening cues in contrast to awareness of externally generated information. First, beliefs about the voices might be regarded as a form of mediator between the content and frequency of the voice and response to it, reflecting underlying core beliefs about self and others (Birchwood, Meaden, Trower, Gilbert & Plaistow, 2000). Second, it has been suggested that voices are experienced as external events because patients fail to recognize internal experiences as belonging to the self (Bentall, 1990). Hence, it is not unreasonable to assume that perceiving a voice as malevolent and commenting on the acts of the patient, such as saying “You are stupid”, might be regarded as a projected automatic thought such as “I’m stupid” or “They think I am stupid”. This influences the information-processing system in similar ways as for people suffering from social anxiety disorder. According to the cognitive model of social anxiety (Clark & Wells, 1995) a shift in attention towards the self leads to impaired performance in the social situations, since fewer attentional resources are used to pay attention towards what is actually happening in the social situation. An interesting extension of the feeling of being harassed by the voice saying things like “You are stupid,”“You can’t not do anything right,” is in a recent review by Paulik (2011) who found after reviewing the literature that patients who experience themselves as of low social rank also experience themselves as being of low rank in relation to the voices. A similar finding was found in another recent review by Hayward, Berry & Ashton (2011. p. 1313) who concluded that “the relationships that hearers develop with their hallucinations share many properties with interpersonal relationships within the social world”.

If a patient perceives the voice as malevolent, the psychological and physiological effects on the individual may be similar to the effects of hostile social relationships in the real world (Gilbert & McGuire, 1998). In hostile relationships attention will thus be directed towards the other person’s verbal and non-verbal behavior, while in patients with auditory hallucinations attention will be directed towards the content and intonation of the voice. Therefore, the attention to external stimuli might become less important for the voice-hearer, and instead he or she might direct more attention towards the voice, especially since the relation to the voice may be close and personal (Benjamin, 1989).

Some of the beliefs about voices can also be regarded as delusions (Chadwick, Birchwood & Trower, 1996), such as the belief that “it is the Devil who wants to punish me.” Moreover, biases in attention can reinforce, maintain or expand delusions, and lead to reduced capacity to acquire and cope with new information (Blackwood, Howard, Bentall & Murray, 2001). Thus, in addition to the finding that beliefs about voices as malevolent affect levels of attention, bias in attention can in turn contribute to a reduced capacity to evaluate the beliefs about the voice, and thereby sustain beliefs about voices as malevolent, and further influence level of inattention, in a somewhat vicious circular process.

We also found that voice frequency (scored from PSYRATS-AHS) affected levels of inattention, independently of whether or not the voice was experienced as malevolent. This is in line with findings of other authors who have described how appraisal processes also are influenced by environmental variables, such as the intensity and duration of the stimuli (Monat & Lazarus, 1991). Further, it has also been described that participants who regarded voices as having a higher power and rank compared to them, were more distressed by the voices, and assessed them as louder and more frequent (Birchwood *et al.*, 2000).

A few of the participants had a formal diagnosis of schizoaffective and delusional disorder, rather than schizophrenia. These patients were nevertheless included in the study since these diagnoses are sub-categories together with schizophrenia in the ICD-10 classification system. Since the purpose of the present study was to investigate the relationship between attention function and auditory hallucinations we actually consider it a strength that a few patients with a sub-category diagnosis were included since it would include commonalities and differences in cognitive deficits across traditional diagnostic borders.

### Clinical implications

The finding that malevolent beliefs about voices affect levels of attention has important clinical implications. This highlights the importance for clinicians to screen patients for their beliefs about voices. Helping voice hearers, within the context of a therapeutic relationship, to change their beliefs and relation to the voices may prove beneficial.

### Limitations

The use of a cross-sectional design may preclude causal inferences. In order to understand further how inattention and auditory hallucinations relate to each other it is essential to measure these variables longitudinally across time. Second, although the patients were diagnosed by experienced clinicians, the inter-rater reliability was not assessed. Moreover, although the attention subscale of SANS has been demonstrated to be a meaningful way to clinically rate schizophrenic patients’ severity of attentional dysfunction (Vadhan *et al.*, 2001), future studies should be replicated utilizing different measures relating to inattention, such as measures of actual interpersonal situations.
